# The influence of factors associated with past reproductive histories on migraines in middle-aged premenopausal women: a nationwide population-based study in Republic of Korea

**DOI:** 10.3389/fneur.2024.1406443

**Published:** 2024-06-10

**Authors:** Seonghoon Kim, Seunghee Na, Young-Do Kim, Dae Woong Bae, Jae Young An, Jeong Wook Park

**Affiliations:** ^1^Department of Neurology, Uijeongbu St. Mary’s Hospital, College of Medicine, The Catholic University of Korea, Seoul, Republic of Korea; ^2^Department of Neurology, Incheon St. Mary’s Hospital, College of Medicine, The Catholic University of Korea, Seoul, Republic of Korea; ^3^Department of Neurology, St. Vincent’s Hospital, College of Medicine, The Catholic University of Korea, Seoul, Republic of Korea

**Keywords:** premenopausal woman, migraine, parity, breastfeeding, oral contraceptives

## Abstract

**Introduction:**

Women can experience various reproductive events, such as pregnancy, childbirth, lactation, and contraception, which cause long-term changes in female hormones. In middle-aged women, the prevalence of migraine is high, and a clear gender difference is evident. This study investigated the effects of factors associated with past reproductive events on the risk of new migraine in middle-aged premenopausal women.

**Methods:**

The influence of reproductive factors on migraine in middle-aged women was investigated using the Korean National Health Insurance Service (KNHIS) and Korean Health Examination (KHE) databases. The reproductive factors of interest were parity, breastfeeding, and oral contraceptive (OC) use. The study included 949,704 middle-aged premenopausal women 40–60 years of age. The study population was divided into two groups based on new diagnosis of migraine during the follow-up period (2009–2018).

**Results:**

The risk of new migraine tended to increase in the primiparous (hazard ratio, HR: 1.179; 95% confidence interval, CI: 1.137–1.221) and multiparous groups (HR: 1.181; 95% CI: 1.142–1.221) compared with the nulliparous group. The breastfeeding ≥12 months group (HR: 1.071; 95% CI: 1.052–1.091) showed a significantly increased risk of new migraine compared with the non-breastfeeding group. All women in the OC groups (< 1 year, HR: 1.048; 95% CI: 1.028–1.069 and ≥ 1 year, HR: 1.100; 95% CI: 1.067–1.134) showed a higher risk of new migraine than those in the non-OC group.

**Conclusion:**

The results of the current study indicate that childbirth, longer breastfeeding, and OC use may be associated with a higher risk of new migraine in middle-aged premenopausal women.

## 1 Introduction

Migraine is a common disabling neurological disorder that imposes a considerable burden on migraine patients and society. In women, migraine prevalence increases rapidly after menarche, reaching 2–3 times that of men in their 20s and 40s, and decreases significantly after menopause (1). Reproductive events, such as pregnancy, childbirth, lactation, contraception, and menstruation, are thought to contribute to migraine prevalence (2).

Lifetime hormonal changes play an important role in female migraine patients. Estrogen is the most important hormonal factor in migraines, and its fluctuation is a main mechanism (3). Estrogen fluctuations decrease during pregnancy and breastfeeding. Plasma estrogen increases more than 30–40-fold during pregnancy compared with levels in non-pregnant women. Estrogen level declines dramatically after childbirth, and a low-estrogen state continues during breastfeeding (3). Clinical observations have demonstrated that migraines tend to improve or cease during pregnancy, mainly in the second and third trimesters (3–5). In the Nord-Trøndelag Health (HUNT2) study, migraine was less prevalent among pregnant women, and the association between migraine and pregnancy was particularly significant for nulliparous women (6). Only a few studies have analyzed the long-term effects of parity, breastfeeding, and other factors associated with childbirth in nonpregnant women. In the HUNT2 study, among all pregnant women, migraines occurred more often in primiparous and multiparous women than in nulliparous women (6).

Oral contraceptives (OCs) are used to prevent pregnancy and modulate the menstrual cycle and are considered an exogenous sex hormone factor. A continuous OC formulation is prescribed for some women to prevent or reduce some forms of migraine, such as menstrual migraine (7). However, negative effects of OCs on migraine have also been reported (8). In a large population-based study, current or previous use of OCs increased the prevalence of migraines in premenopausal women compared with women who never used OCs. Studies have shown inconsistent results on the effects of menopausal hormone therapy (MHT), another exogenous hormonal factor, on migraines. Despite its potentially negative effects, MHT is often recommended for perimenopausal women with migraines (9). We previously reported that MHT might increase the risk of migraines in postmenopausal women (10). Therefore, reproductive factors may hypothetically influence migraine prevalence.

The purpose of the present study was to investigate the effects of factors associated with past reproductive events on the incidence of migraine diagnosis in middle-aged premenopausal women. We used a population-based cohort that included medical information obtained from the Korean National Health Insurance Service (KNHIS) database.

## 2 Material and method

### 2.1 Study population and study design

To investigate the influence of reproductive factors on middle-aged women, we used linked Korean National Health Insurance Service (KNHIS) and Korean Health Examination (KHE) data for the same participants to evaluate the occurrence of migraine. Middle-aged premenopausal women were defined as those aged 40 to 60 years with regular menstruation. The KHE includes a life-transition period health examination at age 40. Therefore, we defined middle age as spanning from 40 to 60 years old. The inclusion criteria of middle-aged premenopausal women were as follows: (1) age 40–60 years, and (2) women with regular menstruation. We excluded women with a history of a previous migraine diagnosis (ICD-10 code) during the washout period (2005–2009). In addition, we excluded participants if they presented with any of the following: (1) menopause, (2) current pregnancy, (3) history of hysterectomy, and (4) history of MHT. Menopause status, current pregnancy, and history of hysterectomy were confirmed from the KHE questionnaire. In this retrospective cohort study, we reviewed migraine occurrence in middle-aged premenopausal women using KNHI data. The KNHIS program provides medical services for the entire Korean population and has a sample size greater than 50 million individuals (11). The study population was divided into the migraine group and control group based on diagnosis of migraine during the follow-up period (2009–2018). We extracted KNHIS claims with ICD-10 codes for migraine and associated medication history. In the KNHIS database, migraine is diagnosed based on the opinions of both migraine-specialists and non-experts from all medical institutions of Korea, which reduces diagnostic accuracy. To increase the diagnostic accuracy, we limited diagnosis of new migraine as at least one event in a woman with migraine diagnosis (ICD-10 codes: G430, G431, G433, G438, G439) and use of medication for migraine treatment (triptan derivatives, ergotamine, topiramate, valproate, propranolol, metoprolol, timolol, flunarizine, amitriptyline, or venlafaxine) during the observation period. We compiled the medication list based on migraine treatment recommendations and medical insurance coverage in Korea (12).

We investigated the associations between reproductive factors of parity number, duration of breastfeeding, and length of OC use and migraine. The duration of breastfeeding was divided into four categories (none, < 6 months, 6–12 months, and ≥ 12 months). The parity number was divided into three categories (0, 1, and ≥ 2). The duration of OC use was divided into four categories based on the total years of OC use (none, < 1 year, ≥ 1 year, and unknown).

### 2.2 Data source and ethics approval

We used the KNHIS and the KHE databases. The KNHIS database includes all claims data for the KNHIS program and lists diagnoses based on International Classification of Disease, Tenth Revision (ICD-10) codes. In Korea, a regular national health examination is mandatory every 2 years for all adults >40 years of age. The health examination items include routine laboratory tests, a work-up for malignancies, physical measurements, and a self-report health questionnaire. All obtained data are stored in the KHE database, which includes data on age, sex, history of comorbid diseases (i.e., hypertension (HBP), dyslipidemia, stroke, ischemic heart disease, and diabetes mellitus (DM)), and health-related behaviors (i.e., alcohol consumption, smoking status, and regular physical activity) for all patients. KHE data include information regarding parity, duration of breastfeeding, and length of OC use. We accessed the KNHIS and KHE database for research purpose. All data were fully anonymized before we access them.

The Institutional Review Board of the Korean National Institute for Bioethics Policy approved this study (NHIS-2023-1-474). Anonymized and de-identified data were used, so informed consent was not required. The Institutional Review Board of The Catholic University of Korea approved this study protocol and waived the need for informed consent (No. UC23ZISI0015). This study was conducted according to the principles of the Declaration of Helsinki.

### 2.3 Statistical analysis

We used Student’s *t*-test for continuous variables and the chi-square test for binary and categorical variables to compare characteristics of groups. Participants were divided based on parity number, length of breastfeeding, and duration of OC use. The incidence rate was calculated as 1,000 person-years in each group, and the hazard ratio (HR) was analyzed using Cox’s proportional hazards regression models with a 95% confidence interval (CI) for each factor relative to the reference. We observed the diagnosis of migraine in each group during the observation period. To control for confounding factors, we used Model 1, which included age, and Model 2, which included age, smoking status, alcohol consumption, physical activity, and body mass index (BMI) as confounders. Model 3 was adjusted for age, smoking status, alcohol consumption, physical activity, BMI, DM, HBP, dyslipidemia, and reproductive factors.

We used Cox’s proportional hazards regression models for each of the four consecutive age intervals (< 45 years, 45–50 years, 50–55 years, and 55–60 years), and the relationship between parity and risk of new migraine was analyzed based on age. The HR and 95% CI in each age group were compared with those of the nulliparous group. The association between duration of breastfeeding and risk of new migraine was analyzed using the same method for all four age groups. To adjust for the effect of parity, we excluded nulliparous women (parity = 0), and parity number was used as a covariate. Statistical analyses were performed using SAS software (version 9.4; SAS Institute, Cary, NC, United States).

## 3 Results

### 3.1 Demographics and characteristics

**Figure 1:**
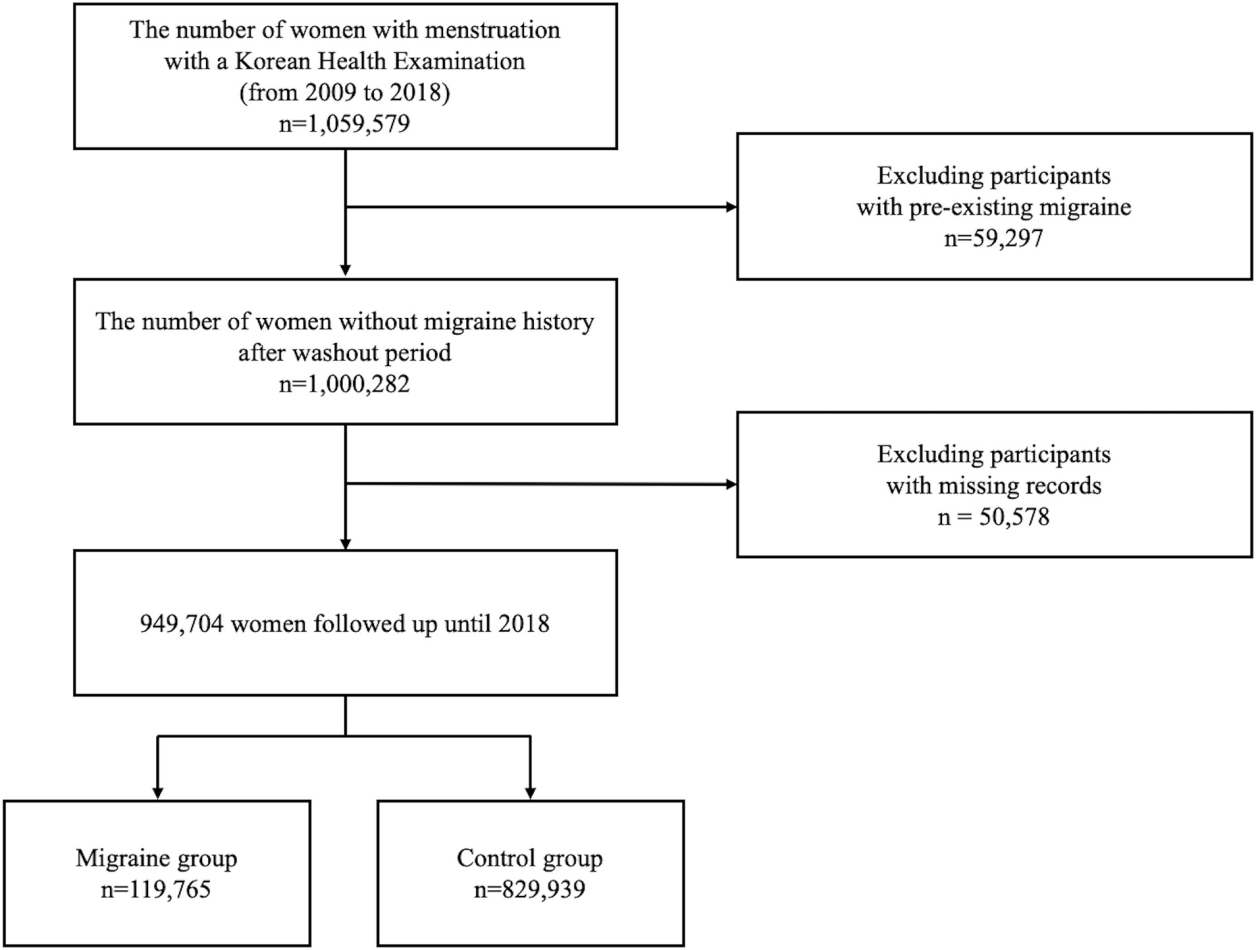
Flow chart of the study process.

From the KHE data from 2009 to 2018, this study included 1,059,579 premenopausal women 40–60 years of age who completed the health examination questionnaire. We excluded 59,292 women with a migraine diagnosis before 2009, and 50,578 were removed due to missing data. Finally, 949,704 premenopausal women without a previous migraine diagnosis were included (Figure 1). The subjects were divided into a migraine group (119,765) and a control group (829,939) based on the presence of a migraine diagnosis during the follow-up period.

The migraine group was older than the control group (migraine vs. control: 45.29 ± 3.94 vs. 45.0 ± 3.96 years, *p* < 0.0001) and showed higher prevalence of HBP, dyslipidemia, ischemic heart disease (*p* < 0.0001), and stroke (*p* = 0.0068) and lower BMI (migraine vs. control: 23.22 ± 3.06 vs. 23.3 ± 3.09 kg/m^2^, *p* < 0.0001) compared with the control group. The prevalence of DM was lower in the migraine group, which also showed larger proportions of current smokers and heavy drinkers (migraine vs. control: current smoker, 3.84% vs. 3.39%; heavy drinker, 1.25% vs. 1.16%; Table 1).

**Table 1:** Demographics and population characteristics based on migraine occurrence.

	Control group	Migraine group	*p*-value
Total	829,939	119,765	
Age, mean ± SD, years	45 ± 3.96	45.29 ± 3.94	<0.0001
Age groups			
<45 years	422,634 (50.92)	56,932 (47.54)	<0.0001
45–50 years	274,970 (33.13)	42,107 (35.16)	
50–55 years	125,365 (15.11)	19,698 (16.45)	
55–60 years	6,970 (0.84)	1,028 (0.86)	
BMI	23.22 ± 3.06	23.3 ± 3.09	<0.0001
HBP	115,586 (13.93)	18,341 (15.31)	<0.0001
DM	25,595 (3.08)	3,407 (2.84)	<0.0001
Dyslipidemia	91,859 (11.07)	14,950 (12.48)	<0.0001
Stroke	1,940 (0.47)	327 (0.55)	0.0068
Ischemic heart disease	3,879 (0.93)	693 (1.16)	<0.0001
Regular physical activity	144,481 (17.41)	20,145 (16.82)	<0.0001
Smoking status			
None	788,392 (94.99)	113,150 (94.48)	<0.0001
Ex-smoker	13,400 (1.61)	2,011 (1.68)	
Current	28,147 (3.39)	4,604 (3.84)	
Alcohol consumption			
None	593,154 (71.47)	86,168 (71.95)	<0.0001
Mild	227,154 (27.37)	32,096 (26.8)	
Heavy	9,631 (1.16)	1,501 (1.25)	

Data are presented as the mean ± standard deviation (SD) or *n* (%). BMI, body mass index; DM, diabetes mellitus; HBP, hypertension.

The proportion of patients with multiparity (parity number ≥ 2) was higher in the migraine group (migraine vs. control; 83.06% vs. 82.25%, *p* < 0.0001). The proportion of patients who were breastfeeding at >12 months was larger in the migraine group than in the control group (migraine vs. control: 33.30% vs. 30.76%). The proportion of subjects with no history of OC use in the control group was higher than in the migraine group (migraine vs. control: never-users, 82.37% vs. 83.3%; Table 2).

**Table 2:** Characteristics of reproductive factors in the control and migraine groups.

	Control group	Migraine group	*p*-value
Parity number			
0	35,803 (4.31)	4,257 (3.55)	<0.0001
1	111,546 (13.44)	16,034 (13.39)	
≥ 2	682,590 (82.25)	99,474 (83.06)	
Duration of breastfeeding			
None	151,998 (18.31)	20,627 (17.22)	<0.0001
<6 months	202,868 (24.44)	27,482 (22.95)	
6–12 months	219,784 (26.48)	31,772 (26.53)	
≥12 months	255,289 (30.76)	39,884 (33.3)	
Duration of OC use			
None	691,314 (83.3)	98,647 (82.37)	<0.0001
<1 year	76,628 (9.23)	11,547 (9.64)	
≥1 year	27,325 (3.29)	4,418 (3.69)	
Unknown	34,672 (4.18)	5,153 (4.3)	

Data are presented as mean ± standard deviation (SD) or *n* (%). OC, oral contraceptive.

### 3.2 Risk of new migraine based on reproductive factors

Table 3 shows the HR with 95% CI for risk of new migraine based on reproductive factors of parity number, duration of breastfeeding, and duration of OC use. After adjusting for confounding factors, we evaluated the risk of new migraine in Model 1 (adjusted for age), Model 2 (adjusted for age, BMI, smoking status, alcohol consumption, and physical activity), and Model 3 (adjusted for age, BMI, smoking status, alcohol consumption, physical activity, DM, HBP, dyslipidemia, and reproductive factors other than the factor to be analyzed). The primiparous (HR: 1.179; 95% CI: 1.137–1.221) and multiparous (HR: 1.181; 95% CI: 1.142–1.221) groups showed a significantly higher risk of new migraine than the nulliparous group. The risk of new migraine was significantly lower in the breastfeeding <6 months group (HR: 0.973; 95% CI: 0.954–0.992) than in the non-breastfeeding group. The risk of new migraine did not significantly differ between the breastfeeding 6–12 months and the non-breastfeeding groups (HR: 1.016; 95% CI: 0.997–1.035). The breastfeeding ≥12 months group showed a significantly higher risk of new migraine than the non-breastfeeding group (HR: 1.071; 95% CI: 1.052–1.091). All OC use groups showed a higher risk of new migraine than the non-OC use group (Table 3).

**Table 3:** Multivariate Cox’s proportional hazards regression model of the relationships between reproductive factors and risk of new migraine.

	Total	Number of migraines	Number of person-years	Incidence rate	HR (95% CI)		
					Model 1	Model 2	Model 3
Parity number							
0	40,060	4,257	353,124.3	12.0552	1 (Ref.)	1 (Ref.)	1 (Ref.)
1	127,580	16,034	1,114,356	14.3886	1.175 (1.136–1.215)	1.182 (1.143–1.223)	1.179 (1.137–1.221)
≥ 2	782,064	99,474	6,828,538	14.5674	1.183 (1.147–1.22)	1.197 (1.161–1.235)	1.181 (1.142–1.221)
Duration of breastfeeding							
None	172,625	20,627	1,511,300	13.6485	1 (Ref.)	1 (Ref.)	1 (Ref.)
<6 months	230,350	27,482	2,017,096	13.6245	0.999 (0.981–1.017)	1.003 (0.985–1.022)	0.973 (0.954–0.922)
6–12 months	251,556	31,772	2,197,394	14.4589	1.042 (1.024–1.06)	1.047 (1.029–1.066)	1.016 (0.997–1.035)
≥12 months	295,173	39,884	2,570,228	15.5177	1.102 (1.083–1.121)	1.104 (1.085–1.123)	1.071 (1.052–1.091)
Duration of OC use							
None	789,961	98,647	6,904,853	14.2866	1 (Ref.)	1 (Ref.)	1 (Ref.)
<1 year	88,175	11,547	768,798	15.0195	1.052 (1.032–1.073)	1.048 (1.028–1.069)	1.048 (1.028–1.069)
≥1 year	31,743	4,418	275,371.3	16.0438	1.114 (1.081–1.148)	1.102 (1.069–1.136)	1.100 (1.067–1.134)
Unknown	39,825	5,153	346,996.4	14.8503	1.027 (0.999–1.057)	1.024 (0.996–1.053)	1.031 (1.003–1.061)

The incidence rates were calculated per 1,000 person-years. Model 1 was adjusted for age. Model 2 was adjusted for age, BMI, smoking status, alcohol consumption, and physical activity. Model 3 was adjusted for age, BMI, smoking status, alcohol consumption, physical activity, DM, HBP, dyslipidemia, and reproductive factors other than the factor to be analyzed. HR, hazard ratio, CI, confidence interval; OC, oral contraceptive; BMI, body mass index; DM, diabetes mellitus; HBP, hypertension.

### 3.3 Age-specific risk of new migraine based on endogenous reproductive factors

**Figure 2:**
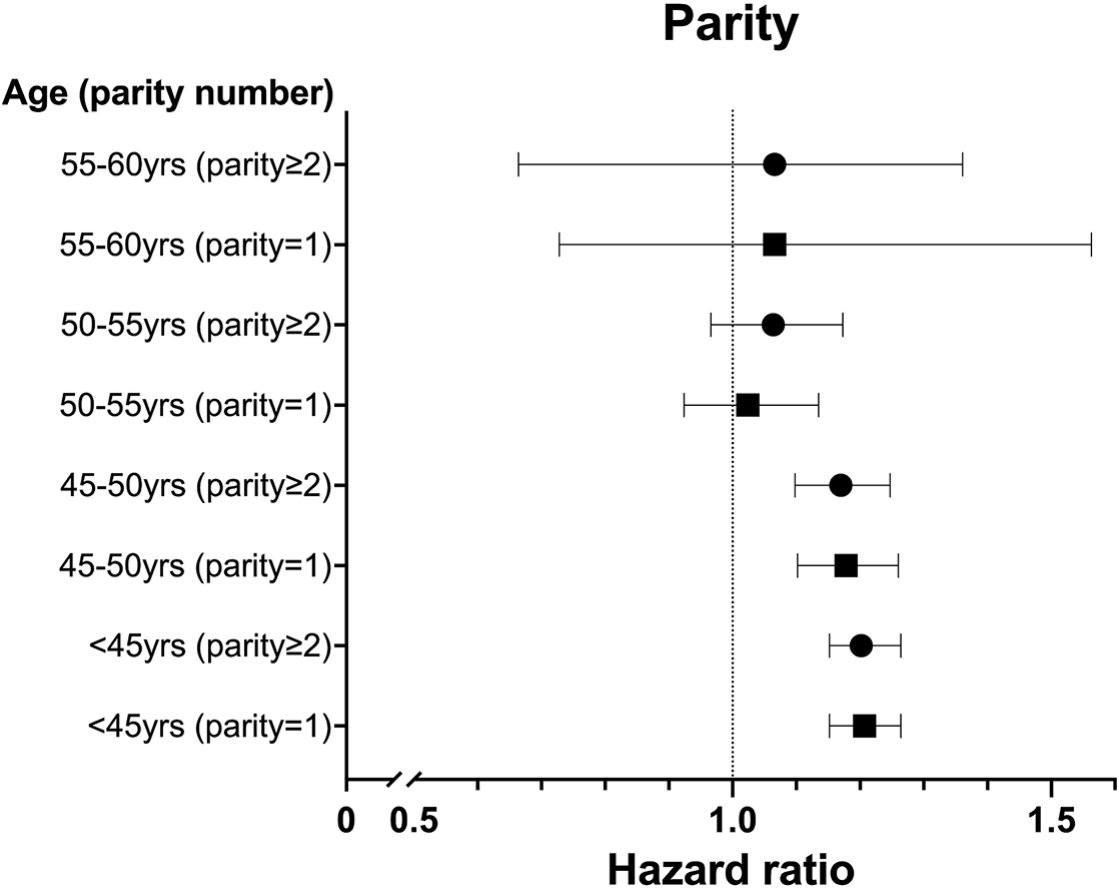
The hazard ratio (HR) for migraine in parous women compared with nulliparous women based on the number of parities in five-year age groups after adjustment for duration of breastfeeding, body mass index (BMI), smoking status, alcohol consumption, physical activity, hypertension, dyslipidemia, and diabetes.

**Figure 3:**
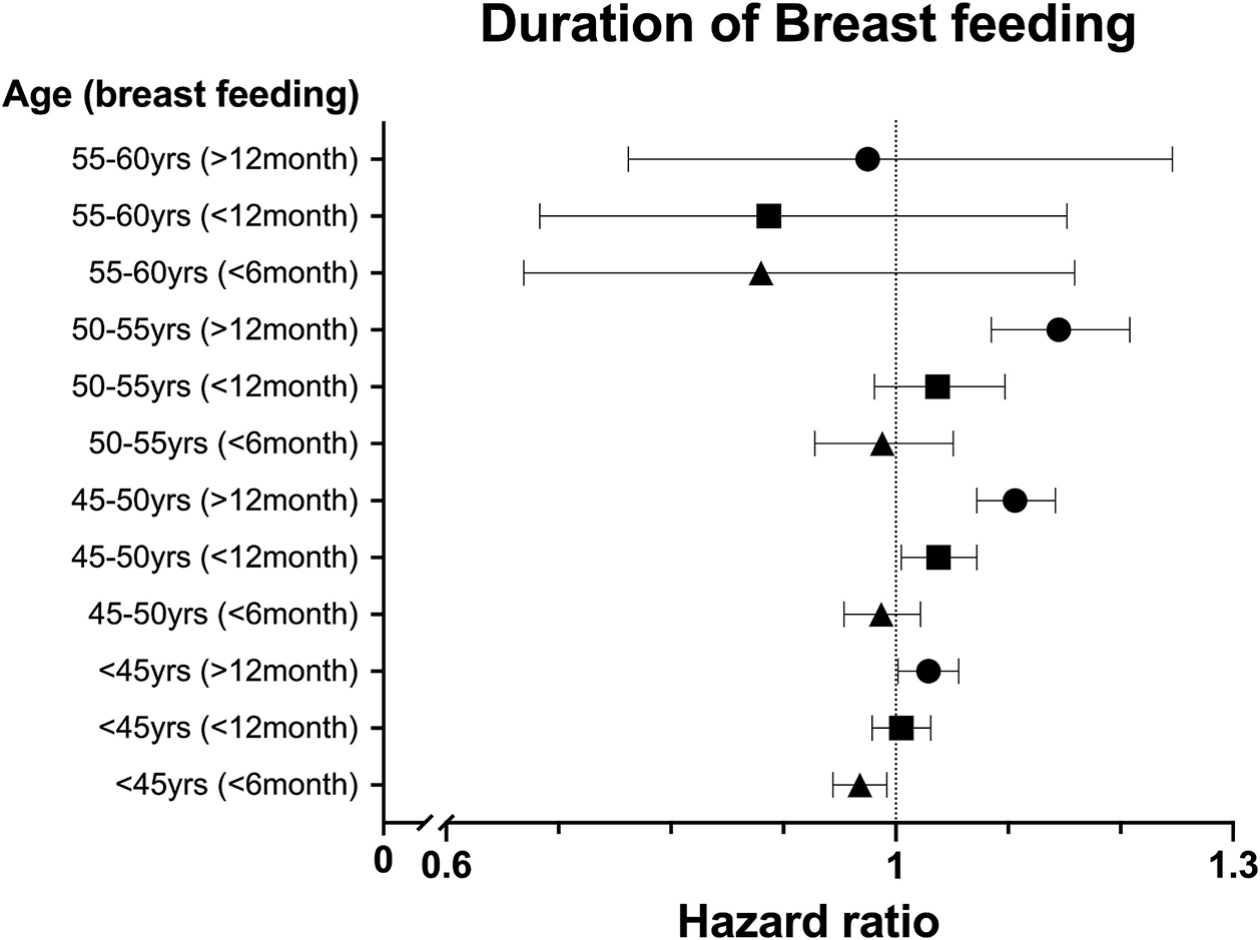
The hazard ratio (HR) for migraine in parous women compared with nulliparous women based on duration of breastfeeding in five-year age groups after adjustment for number of parities, body mass index (BMI), smoking status, alcohol consumption, physical activity, hypertension, dyslipidemia, and diabetes.

We analyzed the HR with a 95% CI association with parity and breastfeeding duration based on age. In the <50 year age group, more than one childbirth experience was associated with a significantly higher risk of new migraine than in the nulliparity group (Figure 2). The age-specific analysis for breastfeeding was performed only in parous women. Breastfeeding >12 months increased the risk of new migraine in the <55 years of age groups, and breastfeeding >6 months increased the risk of new migraine in the <50 years of age groups. In the <45 years of age group, breastfeeding <6 months showed a significantly lower risk of new migraine compared with the non-breastfeeding group (Figure 3).

## 4 Discussion

This was a population-based retrospective cohort study using the KNHIS database. We investigated the relationships between reproductive factors and risk of new migraine in middle-aged premenopausal women. The findings showed that higher parity number, longer duration of breastfeeding, and use of OCs were associated with a higher risk of new migraine in middle-aged premenopausal women.

### 4.1 Parity and migraines

The effect of parity on the course of headaches among women migraine patients remains inconclusive. In two large prospective studies, parity did not influence migraine activity during pregnancy (13, 14). In another prospective study, a significantly lower migraine prevalence was found among only nulliparous pregnant women (6). However, the effects of childbirth on migraine in the long-term, not just during or immediately after childbirth, have rarely been studied. Our study showed that a higher parity number was associated with a higher risk of new migraine, but the mechanism remains unclear.

Socioeconomic factors are significant contributors to migraine prevalence. While migraine rates are elevated in low-income households, epidemiological studies indicate lower prevalence in low-income countries. Disparities in healthcare access, education, and women’s societal status likely contribute to these variations. Moreover, women encounter limitations in migraine treatment options during pregnancy and breastfeeding (15, 16). Childbirth and parenting may be significant physical, emotional, and economical burdens for women. Psychological and emotional stress can exacerbate migraines (17). Therefore, parenting stress may influence the risk of migraine in women. Multiparity and low economic state are associated with high parenting stress (18). Women with children have greater parenting stress than nulliparous women, which can aggravate migraines (19, 20). In our study, the effect of parity on risk of new migraine was not significant in women after 55 years of age. The children of such women tend to be adults, reducing the burden of parenting. However, our study did not investigate the relationships between stress factors and risk of new migraine.

The hormonal state of a patient is thought to play an important role between migraine and parity. The lifetime number of years of menstruation (LNYM) is the total duration of menstruation (excluding pregnancy and breastfeeding), which reflects the cumulative exposure to endogenous estrogen in women (21). A significant inverse relationship exists between LNYM and sex hormone-binding globulin (SHBG), which induces serum estrogen to travel from the circulation into cells. Low serum SHBG results in increased endogenous estrogen exposure, and SHBG is increased in relation to parity number (22). A higher LNYM increases the risk of breast and endometrial cancer, although this risk is decreased with higher parity number (21–24). A significant inverse association between migraine and breast cancer was reported in a meta-analysis (25). We previously found that a shorter LNYM increases migraine prevalence in postmenopausal women (10). Our present study indicated that higher migraine risk was associated with childbirth, which is related to a shorter length of endogenous estrogen exposure. These results indicate that shorter endogenous estrogen exposure might affect the risk of migraine.

In our study, the effects of parity on migraine were not significant in women after 55 years of age. Because the average age at menopause in Korea is 49.3 ± 3.5 years, the women in the >55 years of age group might experience a relatively longer menstruation period over their lifetime (26). This longer endogenous estrogen exposure may influence migraine, but the exact mechanism is unclear. In a previous study, the incidence of migraine in nonpregnant women was lower in nulliparous women than in women with children, but the difference was not significant after 40 years of age, in agreement with the findings of our study (6).

### 4.2 Breastfeeding and migraine

While breastfeeding positively affects migraine activity, the association and long-term effects have not been clearly established. In some studies, breastfeeding was reported to impede recurrence of migraine (14, 27). Migraine recurred within the first postpartum month in 100% of women who did not breastfeed and in 43.2% of those who were breastfeeding (27). In contrast, in a large population-based study, women who experienced migraine during pregnancy and puerperium reported that breastfeeding did not influence migraine occurrence (28). Our results showed that breastfeeding >12 months was associated with a higher risk of new migraine, which was significant in patients <55 years of age. Our study was unique in that we investigated the long-term effects of breastfeeding following childbirth on risk of new migraine in premenopausal women.

Hormonal factors may contribute to the association between breastfeeding and migraine. Breastfeeding inhibits ovulation and maintains low estrogen level with minimal fluctuation (3). A long breastfeeding period shortens the LNYM, which may be associated with a high risk of new migraine. The 55–60 years age group was exposed to relatively longer endogenous estrogen, which may influence migraine. In age-specific analysis, the protective effect of breastfeeding <6 months against migraine was significant in the <45 year age group. However, caution is needed in interpreting this result. In Korea, 7.1% of all childbirths occur in women between 40 and 45 years of age, and these women were not excluded from our study (29). Therefore, it’s possible that women who were breastfeeding or in the immediate postpartum period were included, which might have led to a lower risk of new migraine observed in this group.

Most previous studies have been conducted during the lactation period, and the evidence for the long-term influence of hormonal factors is insufficient. In addition, emotional, physical, and parental stress may have influenced our results. Women with a prolonged lactation period often experience a decrease in sleep quality because of the need for night feeding and the delay in returning to regular daily activities (30). Therefore, various factors should be considered regarding the relationship between breastfeeding and migraine.

### 4.3 OCs and migraine

OCs are frequently used by women for birth control and may exacerbate migraine symptoms (31). In a randomized controlled study, 70% of migraine patients experienced worsening in migraine when taking OCs (32). Headache patterns did not change in 44–67% of migraine patients, became worse in 24–36%, and improved in 5–8% with use of OCs (33–35). A large cross-sectional population study investigated the effects of OCs on the prevalence of migraine in 13,994 premenopausal women and reported that those currently using OCs had a higher prevalence of migraine than subjects who never used OCs (OR: 1.4; 95% CI: 1.2–1.7) (8). We previously reported that MHT increases the risk of migraine in postmenopausal women (10), indicating that the same hormonal factors that cause an interruption in the normal menstruation cycle may also increase the risk of migraine. Various routes of MHT, such as transdermal patches and etonogestrel implants, have been recently implemented (36, 37). A previous study reported that the transdermal patch might improve menstrual migraine (38). However, evidence on the relationship between exogenous hormones and migraine is unclear (39). The results of our study showed that all OC use groups had a higher risk of new migraine than the non-OC use group, and the risk tended to increase based on the length of OC use. Considering the limited data available on the exact type, dose, and persistence of OC usage, the results of our study should be interpreted with caution.

This large population-based study analyzed information from the linked KNHIS and KHE databases. Consequently, there were several limitations. KNHIS data did not include clinical information, so we could not investigate the association between reproductive factors, migraine frequency, recurrence, and severity. We identified only migraine participants who were currently taking migraine-specific abortive and preventive medications and had ICD-10 codes in the KNHIS database. Therefore, it is possible that our study did not identify migraine patients who only took analgesics to treat their migraines. We obtained patient clinical information from the KHE database, which involves some limitations. Information regarding childbirth and breastfeeding at the time of migraine occurrence was not confirmed. Socioeconomic weighting based on the child’s upbringing after birth can play a crucial role but could not be assessed. We were unable to access data on contraceptive methods such as intrauterine devices (IUDs) or implants, beyond oral contraceptives (OCs), which limited our ability to assess their impact in this study. In addition, we could not determine the exact type of OC being used. However, as only combined OCs are available in Korea, the current results reflect the influence of combined OCs. Despite these limitations, our population-based study uses a large sample size, which is considered to produce a significant result. In addition, we presented the remote effects of reproductive factors on migraine.

## 5 Conclusion

Our study revealed that childbirth experiences, longer breastfeeding, and OC use might be associated with a higher risk of new migraine in middle-aged premenopausal women. These results may be helpful for understanding and treating migraine patients. Further studies are needed to clarify the associations between migraine and reproductive factors.

## Data availability statement

The data analyzed in this study is subject to the following licenses/restrictions: raw data are available from the National Health Insurance Date Sharing Service for researchers who meet the requirements for access to confidential data. Requests to access these datasets should be directed to https://nhiss.nhis.or.kr/bd/ab/bdaba000eng.do.

## Ethics statement

The studies involving humans were approved by the Institutional Review Board of the Catholic University of Korea and the Institutional Review Board of the Korean National Institute for Bioethics Policy. The studies were conducted in accordance with the local legislation and institutional requirements. Written informed consent for participation was not required from the participants or the participants' legal guardians/next of kin in accordance with the national legislation and institutional requirements.

## Author contributions

SK: Formal analysis, Investigation, Methodology, Writing – original draft. SN: Writing – review & editing. Y-DK: Writing – review & editing. DB: Formal analysis, Methodology, Writing – review & editing. JA: Writing – review & editing. JP: Conceptualization, Funding acquisition, Project administration, Supervision, Writing – review & editing.
